# Perinatal mental health around the world: priorities for research and service development in Africa

**DOI:** 10.1192/bji.2020.16

**Published:** 2020-08

**Authors:** Mwawi Ng'oma, Tesera Bitew, Malinda Kaiyo-Utete, Charlotte Hanlon, Simone Honikman, Robert C. Stewart

**Affiliations:** 1 Doctoral Fellow, African Mental Health Research Initiative, Department of Mental Health, College of Medicine, University of Malawi, Malawi; 2 Post-Doctoral Fellow, African Mental Health Research Initiative, Department of Psychiatry, School of Medicine, College of Health Sciences, Addis Ababa University, Ethiopia; 3 Doctoral Fellow, African Mental Health Research Initiative, Department of Psychiatry, College of Health Sciences, University of Zimbabwe, Harare, Zimbabwe; 4 Reader in Global Mental Health, Centre for Global Mental Health, Institute of Psychiatry, Psychology and Neuroscience, King's College London, UK; 5 Associate Professor, Department of Psychiatry and Mental Health, University of Cape Town, South Africa; 6 Senior Clinical Research Fellow, Division of Psychiatry, University of Edinburgh, Scotland. Email: robert.c.stewart@ed.ac.uk

**Keywords:** Low- and middle-income countries, perinatal psychiatry, transcultural psychiatry

## Abstract

Africa is a diverse and changing continent with a rapidly growing population, and the mental health of mothers is a key health priority. Recent studies have shown that: perinatal common mental disorders (depression and anxiety) are at least as prevalent in Africa as in high-income and other low- and middle-income regions; key risk factors include intimate partner violence, food insecurity and physical illness; and poor maternal mental health is associated with impairment of infant health and development. Psychological interventions can be integrated into routine maternal and child healthcare in the African context, although the optimal model and intensity of intervention remain unclear and are likely to vary across settings. Future priorities include: extension of research to include neglected psychiatric conditions; large-scale mixed-method studies of the causes and consequences of perinatal common mental disorders; scaling up of locally appropriate evidence-based interventions, including prevention; and advocacy for the right of all women in Africa to safe holistic maternity care.

In terms of sheer scale, the health of mothers in Africa – including their mental health – will be one of the major global health challenges of the 21st century: Africa is home to 1.25 billion people, but this figure is predicted to rise to as many as 4.5 billion by 2100. Africa is a continent of 54 countries with diverse – and rapidly changing – cultures and socioeconomic circumstances, both between and within countries. Understanding and responding to this rich and complex situation brings both challenges and opportunities for maternal mental health researchers, practitioners and policy makers in Africa. This paper highlights recent epidemiological findings, gives examples of service innovations and intervention research, and outlines future priorities for research, service development and advocacy.

## Presentation, prevalence, causes and consequences

Over the past 15 years there has been an increase in published research on maternal mental health in Africa, building on pioneering studies such as those conducted by John Cox in Uganda.^1^ Qualitative studies show that perinatal mental health problems are recognised in communities as important and disabling; they are often seen as understandable responses to psychosocial stress rather than as ‘health’ problems. Pregnancy is regarded as a period of marked uncertainty, when women are vulnerable to physical, financial, interpersonal, supernatural and spiritual jeopardy. Respondents in Ethiopia referred to pregnancy as a period ‘between life and death’.^2^

Most quantitative studies have focused on perinatal common mental disorders (CMDs), particularly depression. Prevalence studies indicate that perinatal CMDs are at least as common in Africa as in high-income and other low- and middle-income regions; a systematic review reported mean weighted prevalences of antenatal and postnatal depression of 11.3 and 18.3% respectively, and of antenatal and postnatal anxiety of 14.8 and 14%.^3^

Risk factors for perinatal CMDs identified in African studies are similar to those found across low- and middle-income countries.^4^ Intimate partner violence appears to be a particularly potent stressor.^5^ Other key risk factors include lack of a confiding relationship, food insecurity and physical illness in the mother (e.g. HIV, obstetric fistula) and infant.

Mental health problems in the perinatal period can affect maternal functioning (particularly in the context of additional adversity) and have been associated with impaired intrauterine and postnatal infant development. In the African literature, perinatal CMDs have been shown to be a risk factor for prolonged labour, delayed breastfeeding initiation^6^ and increased frequency of infant febrile and diarrhoeal episodes. Some studies have shown an association between perinatal CMDs and impaired foetal/infant growth, although others have not. Whether this reflects true differences between settings or methodological factors remains unclear.^7^

Perinatal mental health problems other than depression remain underresearched in Africa: these include postpartum psychosis, suicide, anxiety disorders and post-traumatic stress disorder (PTSD). The latter is likely to be a significant problem, given the high rates of obstetric (and other) traumatic exposures in many African settings.^8^ Alcohol use in pregnancy is a major public health problem in some countries, particularly South Africa, which has the highest global rate of foetal alcohol syndrome.^9^

## Promotion, prevention, identification and intervention

Evidence from across the globe indicates that a multilevel approach to tackling maternal mental health problems is required: health promotion and primary/secondary prevention efforts in community and healthcare settings; identification and management integrated into routine maternal and child healthcare; and access to specialist mental health services that can meet the particular needs of mothers. Barriers to implementation in Africa include: stigmatising beliefs and lack of awareness in both communities and healthcare institutions; low prioritisation of mental health in government health budgets, resulting in a critical shortage of human resource; and existing psychiatric services that are institution based and localised in urban settings. As a consequence, a huge ‘treatment gap’ exists in most African countries between those women who would benefit from intervention and those who can access it.

Randomised controlled trials (RCTs) from a number of African countries have shown that psychosocial interventions provided by supervised non-specialised workers can be integrated into routine maternal and child healthcare services, although the optimal model and intensity of intervention remain unclear and are likely to vary across settings. In a small study in an urban primary care clinic in Zimbabwe, Chibanda et al found that group problem-solving was more effective than pharmacotherapy in reducing depressive symptoms among postnatal women.^10^ In Nigeria, Gureje et al compared high-intensity and low-intensity psychosocial interventions for perinatal depression delivered by trained community midwives.^11^ The interventions were feasible and acceptable, and both groups had high rates of symptom resolution. However, there was no difference in effectiveness between the high- and low-intensity interventions overall. In a South African study conducted in an economically deprived peri-urban settlement in Cape Town, Lund et al compared a six-session psychosocial intervention with enhanced usual care.^12^ There was no effect of the intervention on the primary depression outcome measure. The authors speculated that the intervention may not have been sufficiently intensive or that enhanced usual care may itself have been effective treatment. In addition, they noted that psychological interventions that focus on developing resilience do not tackle important upstream risk factors such as poverty and family and community violence.

Other interventions have integrated maternal mental healthcare into community child development programmes. In a cluster RCT, Singla et al evaluated a parenting intervention to address maternal psychological well-being and child development in rural Uganda.^13^ This was conducted among mothers of children aged 12–36 months, but the principles could apply to a perinatal population. The intervention was delivered by community volunteers and included spousal involvement. Mothers in the intervention group had reduced depressive symptoms and their children attained higher cognitive and language development.

An example of a successful clinical service is the Perinatal Mental Health Project (PMHP, www.pmhp.za.org) in Cape Town, South Africa. Some key learning points from an evaluation of the PMHP were: screening instruments should be brief, binary (i.e. yes/no answers) and locally validated; an additional cadre of clinic staff (mental health counsellors) with particular interest and training may be needed; and adequate clinical supervision and access to referral are required.^14^ A novel feature of the PMHP model has been recognition that, to promote the delivery of more psychologically oriented empathic care, the intervention should also address the well-being of the care providers themselves.

Women who experience severe mental illness (SMI) in the perinatal period (e.g. postpartum psychosis, severe depression, bipolar disorder, schizophrenia) have treatment needs that are particularly challenging to meet in environments with low healthcare capacity and resources. Guidelines are lacking for safe perinatal prescribing in contexts of limited drug availability and where cost, safety and cultural issues often prohibit formula feeding. There is a lack of evidence in Africa regarding appropriate care pathways for perinatal SMI; task-shared models of care, whereby primary care and maternal care health workers provide frontline care for women with severe mental illness, are being tested in the African setting but rigorous evaluation is needed to ensure safety as well as clinical benefit.

## Future research and service development priorities

Given the scale of the problem, there remains a dearth of maternal mental health research in Africa. There is a need to expand capacity and activity in both observational and intervention research. There should be an extension of focus to include neglected areas such as anxiety, PTSD, substance misuse and SMI in the perinatal period.

A priority for future observational research is to understand the risk, resilience and impact of maternal CMDs in the context of diverse but rapidly changing societies. A range of methodological approaches is required, including anthropological studies, economic impact analyses and large longitudinal epidemiological studies (such as the Generation Malawi study, funded by the UK's Medical Research Council and Wellcome Trust), to enable investigation of the interaction between socioeconomic, interpersonal and genetic factors. This research can inform the development of evidence-based prevention and health promotion approaches applicable in African contexts.

Intervention development research should build on the evidence to date to determine whether particular approaches offer advantages in terms of acceptability, effectiveness and cost-effectiveness (e.g. different theoretical models; delivery by general maternal and child healthcare staff versus introduction of additional mental health practitioners; individual, family or group interventions; medication protocols; universal versus targeted approaches). The importance of targeting interventions to specific risk areas, such as intimate partner violence, poverty, nutrition and mother–child interaction, should be investigated.

Implementation science and health services research should be used to drive the scaling up of effective interventions for both perinatal CMDs and SMI. Successful integration of mental healthcare into routine maternal and child healthcare services will require locally appropriate treatment guidelines, supervision structures and referral pathways, and training of specialist mental healthcare providers in service planning, education and advocacy roles.^15^ Service development should be evidence based and driven by a commitment to the belief that all women have a right to safe holistic maternity care and, when needed, access to mental health services that are non-discriminatory and promote recovery of their maternal role.

Most of the research focus to date has been on interventions delivered in the healthcare system context. Given the role of risk factors arising from economic and gender-based inequalities, it is also important to explore promotion/prevention strategies in other arenas, such as education, poverty reduction and human rights.

## Conclusions

This ambitious research and service development agenda requires an urgent scaling up of research and clinical capacity on the continent, as set out in the World Psychiatry Association's position statement on perinatal mental health.^16^ Multicountry consortia such as the African Mental Health Research Initiative (AMARI) are an effective approach to meeting this objective. Partnership between research institutions, policy makers, practitioners and patients is needed to ensure that innovations are locally appropriate and sustainable. There is a need to include maternal mental health in policies, guidelines, educational curricula and relevant legislation. Collaborations such as the African Alliance for Maternal Mental Health (AAMMH, www.aammh.org) and the International Marcé Society for Perinatal Mental Health (https://marcesociety.com) are creating networks of organisations and individuals dedicated to these aims. Alongside these initiatives, there is a need for sustained investment if Africa is to meet the mental health needs of its mothers now and in the coming century.

## Supplementary material

For supplementary material accompanying this paper visit http://dx.doi.org/10.1192/bji.2020.16. click here to view supplementary material
